# Morphometry and Morphology of the Fovea Capitis of the Femoral Head and its Associated Implications

**DOI:** 10.7759/cureus.79992

**Published:** 2025-03-03

**Authors:** Rajani Singh, Nisha Yadav

**Affiliations:** 1 Anatomy, Uttar Pradesh University of Medical Sciences, Saifai, IND

**Keywords:** acetabulum, femoral head, fovea capitis, hip joint, joint pathology

## Abstract

Background and objective: The fovea capitis, a depression in the posteroinferior quadrant of the head of the femur, provides attachment to the ligament of the head of the femur, transmitting vessels to the head of the femur. The ligament is often disrupted, leading to avascular necrosis of the head of the femur. The morphometry and morphology of the head of the femur and fovea capitis are useful in various clinical conditions involving the hip joint. The study aims to assess the morphology and morphometry of the head of the femur and fovea capitis and to correlate these with clinical implications.

Methods: The study was conducted in the Department of Anatomy, Uttar Pradesh University of Medical Sciences (UPUMS), Saifai, India, using 40 femora of unknown age and sex. The morphometry was done using a digital vernier caliper, and morphological forms were observed.

Results: The mean perimeter of the femoral head was 12.3 ± 1.09 cm, and that of the fovea capitis was 4.2 ± 0.9 cm. The area of the femoral head was 48.27 ± 1.02 cm², and that of the fovea capitis was 4.84 ± 0.1 cm². The most common morphological form was oval-shaped, constituting 50%, followed by circular and triangular shapes. The least common morphological form observed was irregular-shaped.

Conclusion: The information provided in this study will be of utmost use in dealing with hip joint pathologies like dysplasia and osteoarthritis, reconstructing injured ligaments of the head of the femur, proximal femur measurements, radiological interpretation, arthroscopic procedures, and other surgical interventions involving the hip joint.

## Introduction

The fovea capitis is a depressed space postero-inferior to the center of the head of the femur [1]. It provides attachment to the ligament of the head of the femur, also known as the ligamentum teres femoris, which forms a channel for the passage of vessels irrigating the femoral head. There is a disruption of blood supply to the femoral head if the ligamentum teres femoris, through which blood vessels are passing to the femoral head, is injured in cases of hip fractures and dislocations, culminating in avascular necrosis of the femoral head [2].

Various dimensions, along with the gross features of the fovea capitis, are essential to conserve or restore the damaged ligament of the head of the femur [3, 4]. The morphometry and morphology of the fovea capitis have a direct impact on the occurrence of dysplasia and osteoarthritis of the hip joint [5, 6]. Therefore, the gross appearance of the fovea capitis may provide important clues for assessing hip joint osteoarthritis and the ligament of the head of the femur. In addition, if there is a fracture in the neck of the femur, then the position of the fovea capitis changes with respect to the normal position; thus, the knowledge of the precise location of the fovea capitis is useful in establishing the rotational position of the head of the femur in fractures of the neck of the femur [7].

Thus, the morphology and morphometry of the fovea capitis are of utmost importance for proximal femur measurements, radiograph interpretation, arthroscopic surgery, and surgical intervention involving the hip joint [8-10]. Moreover, the morphology of the fovea capitis is very useful for anthropologists to determine the sex of an individual and differentiate the femur from other long bones [11]. Considering the immense clinical and anthropological importance of the fovea capitis, the study was conducted.

The aim of the study is to elucidate the morphometry and morphological features of the fovea capitis and to correlate these parameters clinically.

## Materials and methods

The study was conducted in the Department of Anatomy, Uttar Pradesh University of Medical Sciences (UPUMS), Saifai, in Uttar Pradesh, India. Forty femurs of unknown age and sex, obtained from the osteology lab of the Department of Anatomy, were used for the current study.

The perimeter of the femoral head and fovea capitis was measured using vernier calipers. Then, the radius of the femoral head and fovea capitis were calculated using the following formula: Perimeter = 2πr, where r is the radius of the head and fovea capitis of the femur.

After that, the area of the femoral head and fovea capitis of the head of the femur were calculated using the formula 4πr². The radius of the femoral head and the radius of the circular-shaped fovea capitis were measured. In addition to this, the transverse and vertical diameters of the oval-shaped fovea capitis and the base and vertical distances of the triangular-shaped fovea capitis were also measured. Then, the mean of the area and various dimensions of the femoral head and fovea capitis located over the head of the femur were calculated using Microsoft Excel 2019 software (Microsoft Corp., Redmond, WA).

Various morphological forms, consisting of oval, round, and triangular-shaped fovea capitis, were observed by visual inspection. Ten femurs with damaged and pathologically deformed femoral heads and fovea capitis were excluded from the study.

Ethical statement

The study was conducted on dry femur bones available in the osteology lab of the university and did not involve live human beings or tissue from live persons; hence, consent was not a requirement.

## Results

The fovea capitis was observed in all 40 femoral heads. The mean perimeter of the femoral head was 12.3 ± 1.09 cm, and that of the fovea capitis was 4.2 ± 0.9 cm. The mean radius of the femoral head was 1.96 ± 0.2 cm, while the mean radius of the fovea capitis was 0.67 ± 0.1 cm.

The mean base of the triangular-shaped fovea capitis was 13.11 ± 1.05 cm, and the vertical distance of this type of fovea capitis was 12.71 ± 0.9 cm. The area of the femoral head was 48.27 ± 1.02 cm², and that of the fovea capitis was 4.84 ± 0.1 cm². The mean percentage area of the fovea capitis in relation to the femoral head was 10.02%.

**Figure 1:**
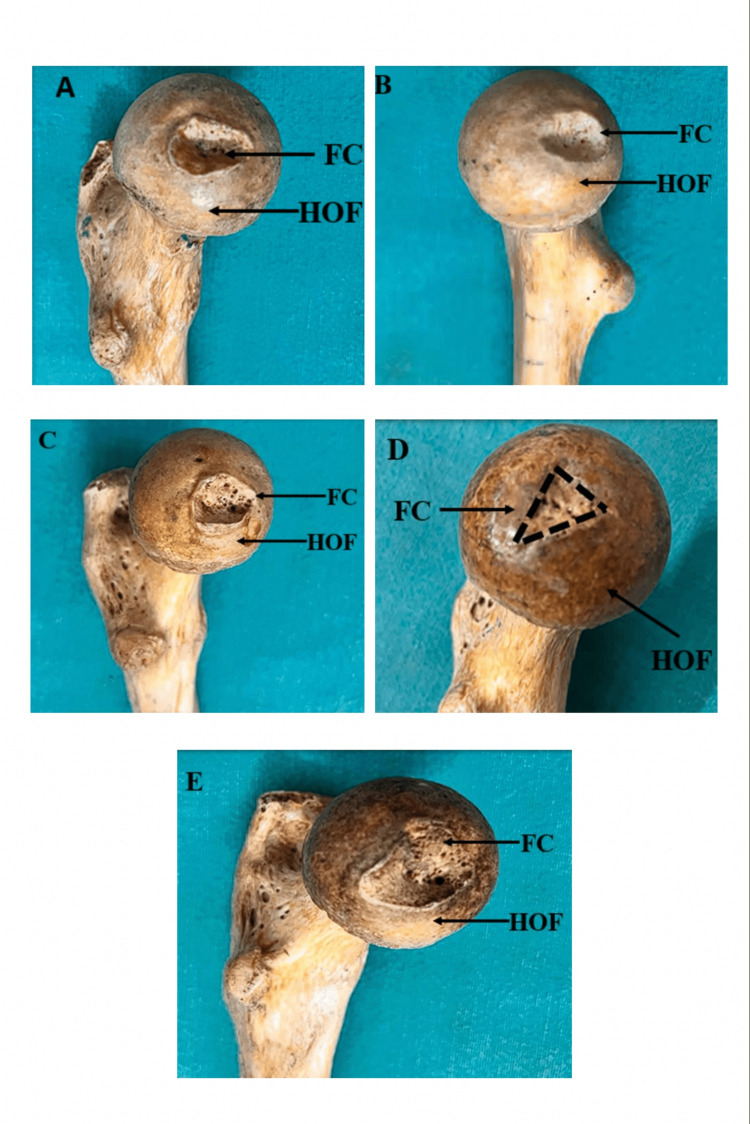
Various morphological forms of fovea capitis A) Horizontal oval-shaped; B) Vertical oval-shaped; C) Circular-shaped; D) Triangular-shaped; E) Irregularly shaped FC: fovea capitis; HOF: head of femur

The various morphological forms observed of the fovea capitis were oval, circular, triangular, and irregular-shaped (Figure 1).

The most common morphological form was oval-shaped, which was observed in 20 femoral heads, constituting 50% of the samples. This was followed by circular and triangular shapes. The least common morphological form observed was irregular-shaped, which was found in one femoral head, constituting 2.5% of the sample size.

Circular-shaped fovea capitis was observed in 14 femoral heads and triangular-shaped fovea capitis in one femoral head. Therefore, the percentages of circular and triangular shapes of the fovea capitis were 35% and 12.5%, respectively.

## Discussion

The morphology and morphometry of the fovea capitis are essential during surgical interventions around the hip region. The perimeter of the head of the femur and the fovea capitis is important for the reconstruction of the hip joint. The mean perimeters of the head of the femur and the fovea capitis are 12.3 cm and 4.2 cm, respectively. There is no literature mentioning the perimeters of the head of the femur and the fovea capitis to compare our results.

Area of the fovea capitis

The area of the fovea capitis and the head of the femur were found to be 144.43 mm² (1.44 cm²) and 1587.17 mm² (15.87 cm²) [12]. In another study, these parameters were 1.8 cm² and 10.5 cm², respectively [13]. However, the area of the fovea capitis and the femoral head was observed to be 171.51 mm² (1.72 cm²) and 1475.35 mm² (14.75 cm²)[2]. In the present study, the area of the fovea capitis is calculated to be 4.84 cm², and that of the femoral head is 48.27 cm². It is clear from the above description that the areas of both the fovea capitis and the femoral head were higher in our study compared to all aforementioned studies. This discrepancy may be attributed to methodological differences used by various investigators [2]. Bertsatos et al. calculated the area of the fovea capitis separately in male and female femora and found that the area of the fovea capitis is higher in males compared to females (219 mm² vs. 177 mm²) [14].

Location of the fovea capitis

In most studies, the fovea capitis was observed to be located in the posteroinferior quadrant of the head of the femur [2, 4, 12, 15]. In the present study, the fovea capitis was also detected in the posteroinferior quadrant of the head of the femur in the majority of our samples, confirming the findings in the literature.

Morphological forms of the fovea capitis

Various morphological forms of the fovea capitis may be circular, oval, triangular, rectangular, square, and irregular.

The ligament of the head of the femur has been designated various names, such as the round ligament or ligamentum teres, as it is ovoid or rounded in configuration at its attachment to the fovea capitis [16]. Different shapes of the fovea capitis have been observed in studies in the literature, which are elaborated on in the succeeding paragraphs.

The most common morphological form of the fovea capitis observed was oval-shaped in 65.6%, followed by circular-shaped in 28% and triangular-shaped in 6.4% in one study [13].

In another study, the shape of the fovea capitis was determined based on the femoral index [11]. According to this criterion, if the femoral index is more than 85, the shape of the fovea capitis is identified as oval-shaped or round-shaped, and if the femoral index is less than 70, the fovea capitis is designated as irregular-shaped or triangular-shaped. Based on this methodology, the authors detected four morphological forms, with the most common shape observed being oval-shaped in 39.73%, followed by round-shaped in 37.67%, and triangular-shaped in 11.64%. The least common variety observed by these investigators was piriform-shaped in 10.96% of cases [11].

In a recent study by Gölpınar, the oval-shaped fovea capitis was the most common morphological form detected in 43.80% of cases, followed by the round-shaped with an incidence of 40.40%. Less common shapes of the fovea capitis observed were triangular and piriform, with incidences of 10.50% and 5.30%, respectively [2].

In the present study, various morphological forms of the fovea capitis found were oval, circular, triangular, and irregular-shaped, with the oval-shaped being the most common type, followed by circular and triangular types. The least common variety of the fovea capitis was the irregular type. The incidences of oval, circular, triangular, and irregular varieties were 50%, 35%, 12.5%, and 2.5%, respectively. Our observation of different morphological forms of the fovea capitis is in confirmation with the aforementioned studies, with the difference that in the current study, no piriform shape of the fovea capitis was detected in contrast to previous studies.

Clinical implications

The ligament of the head of the femur, which is attached to the fovea capitis, forms a conduit for the vessels irrigating the head of the femur. This ligament is often injured during hip fractures and dislocations, disrupting the blood supply to the head of the femur and culminating in avascular necrosis of the femoral head [2].

The morphometry and morphology of the fovea capitis are of paramount importance for surgical interventions to preserve and reconstruct the damaged ligament of the head of the femur due to the intimate association between the fovea capitis and the ligament of the femoral head [3, 4].

There is a definite correlation between the morphometry and location of the fovea capitis with the occurrence of dysplasia and osteoarthritis in the hip joint [5, 6]. Various morphological forms of the fovea capitis may provide clues to hip joint osteoarthritis and the status of the ligament of the femoral head. In addition, knowledge of the location of the ligament of the head of the femur may help estimate the rotational position of the femoral head in fixation approaches in femoral neck fractures [7, 17], for interpreting radiological images, and for diagnostic hip arthroscopy in cases with a missing or disrupted ligament of the head of the femur [13].

Thus, the fovea capitis is a salient anatomical entity for proximal femur measurements, radiological interpretation, arthroscopic procedures, and surgical interventions involving the hip joint [8-11]. Moreover, morphological information related to the fovea capitis is essential for ascertaining gender and differentiating the femur from other long bones in anthropology [11].

Limitations of the study

The study's limited sample size of 40 femurs may not be representative of the general population. The femurs' unknown age and sex introduce variability in measurements and morphology. The study did not directly investigate clinical correlations with hip joint conditions.

## Conclusions

We estimated the perimeter, area of the femoral head and fovea capitis, location, and morphological forms of the fovea capitis in the current study. The most common location of the fovea capitis was the posteroinferior quadrant of the head of the femur, similar to findings in the literature. The areas of the fovea capitis and the femoral head were higher than those calculated by other investigators. Regarding the morphological forms of the fovea capitis, oval types were the most common, followed by round, triangular, and irregular types, in confirmation with studies in the literature.

The information provided in this study is of utmost importance in dealing with hip joint-related pathologies such as dysplasia and osteoarthritis, reconstruction of the injured ligament of the head of the femur, proximal femur measurements, radiological interpretation, arthroscopic procedures, and other surgical interventions involving the hip joint. The observed variations in the morphology of the fovea capitis emphasize the need for further research to establish standardized measurement techniques and to explore the clinical implications of these variations.
